# Systematically Characterizing A-to-I RNA Editing Neoantigens in Cancer

**DOI:** 10.3389/fonc.2020.593989

**Published:** 2020-12-10

**Authors:** Chi Zhou, Zhiting Wei, Liye Zhang, Zhaoyi Yang, Qi Liu

**Affiliations:** ^1^ Translational Medical Center for Stem Cell Therapy and Institute for Regenerative Medicine, Shanghai East Hospital, Bioinformatics Department, School of Life Sciences and Technology, Tongji University, Shanghai, China; ^2^ School of Life Science and Technology, ShanghaiTech University, Shanghai, China; ^3^ Department of Pharmacy, The First Affiliated Hospital of University of Science and Technology of China, Hefei, China

**Keywords:** cancer immunotherapy, neoantigen identification, RNA editing, melanoma, predictive biomarker

## Abstract

A-to-I RNA editing can contribute to the transcriptomic and proteomic diversity of many diseases including cancer. It has been reported that peptides generated from RNA editing could be naturally presented by human leukocyte antigen (HLA) molecules and elicit CD8+ T cell activation. However, a systematical characterization of A-to-I RNA editing neoantigens in cancer is still lacking. Here, an integrated RNA-editing based neoantigen identification pipeline *PREP* **** (P****rioritizing of R****NA ****Editing-based ****Peptides) was presented. A comprehensive RNA editing neoantigen profile analysis on 12 cancer types from The Cancer Genome Atlas (TCGA) cohorts was performed. **** *PREP* was also applied to 14 ovarian tumor samples and two clinical melanoma cohorts treated with immunotherapy. We finally proposed an RNA editing neoantigen immunogenicity score scheme, *i.e. REscore*, which takes RNA editing level and infiltrating immune cell population into consideration. We reported variant peptide from protein IFI30 in breast cancer which was confirmed expressed and presented in two samples with mass spectrometry data support. We showed that RNA editing neoantigen could be identified from RNA-seq data and could be validated with mass spectrometry data in ovarian tumor samples. Furthermore, we characterized the RNA editing neoantigen profile of clinical melanoma cohorts treated with immunotherapy. Finally, *REscore* showed significant associations with improved overall survival in melanoma cohorts treated with immunotherapy. These findings provided novel insights of cancer biomarker and enhance our understanding of neoantigen derived from A-to-I RNA editing as well as more types of candidates for personalized cancer vaccines design in the context of cancer immunotherapy.

## Introduction

Cancer immunotherapy strategies including adoptive T-cell transfer (ACT) with Chimeric antigen receptor T-cell (CAR-T) or Tumor Infiltrating T-cell (TIL), cancer vaccine and immune checkpoint blockade with anti-CTLA4/anti-PD1 inhibitors have exhibited tremendous clinical power in cancer treatment (1–3). These therapy strategies relied on tumor-specific neopeptides (so-called neoatigens) which are recognized by tumor cytolytic T-cells (4). Neoantigens that arise in cancer cells result from non-synonymous genomic mutations such as SNVs and INDELs. The landscape of somatic neoantigen of pan-cancer and cohorts treated by immune checkpoint inhibitor has been well-characterized, and tumor mutational load and neoantigen load were demonstrated to be strongly correlated with the response to immune checkpoint blockades in several cancer types (2, 3, 5).

Protein variant could also arise from transcriptome changes. Retained intron, one type of RNA splicing, resulting from splicing errors which lead to inclusion of intron in mRNA transcript has been demonstrated to be a source of neoantigens in cancer (6). RNA editing (RE) is a common post-transcriptional modification that alters specific nucleotides in RNA sequences, which can also lead to non-synonymous substitutions and generate novel protein (7, 8). The A-to-I [detected as adenosine-to-guanosine (A to G) mismatches in transcriptome] RE (hereinafter referred to as RE) is the most common type of RE in human. It is catalyzed by the adenosine deaminases that act on RNA (ADARs) family of enzymes, which bind double-stranded RNA (dsRNA) and transform adenosines into inosines at specific positions. Three members of this family including ADAR1, ADAR2, and ADAR3 are encoded in the genome. ADAR1 and ADAR2 are expressed ubiquitously and responsible for the majority of editing activity, while ADAR3 expressed mainly in the brain at a low level is catalytically inactive. A-to-I RE could contribute to the transcriptomic and proteomic diversity of many diseases including cancer (9). It has been reported that elevated RE may facilitate the specific autoimmune disease, *i.e.*, Systemic Lupus Erythematosus (SLE) progression by increasing autoantigen burden (10). Evidence has been provided that peptides resulting from A-to-I RE could be naturally presented by human leukocyte antigen (HLA) molecules, demonstrated that RE extends the classes of HLA presented antigens and that these cancer antigens can be recognized by CD8^+^ T cells (11). However, systematical characterization of neoantigens derived from RE is still lacking for the cancer community. It would be intriguing to investigate the landscape of RE neoantigens in different cancer types and analyze their correlations related to tumor immunogenicity, patients’ clinical covariates and clinical benefit from immunotherapy.

## Materials and Methods

### Design of Prioritizing of A-to-I RNA Editing Peptides

**Figure 1:**
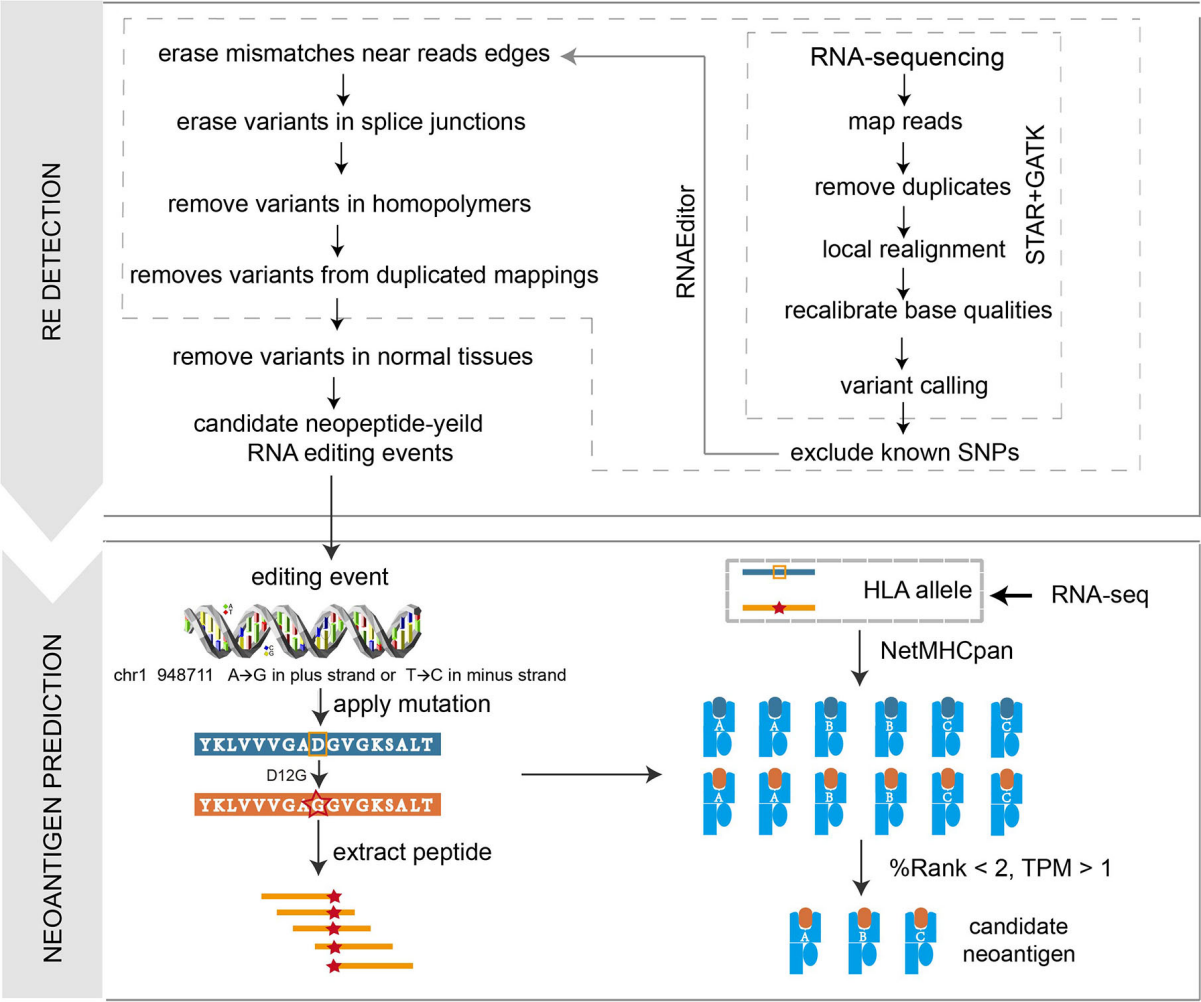
Illustration of the *PREP* workflow. In the *PREP* pipeline, RE sites are first identified and filtered, then the variants are applied to the proteomic level to obtain mutant peptides, which are cut into 9–11-mer short peptides. NetMHCpan 4.0 is utilized to predict the binding affinity between the peptide and given HLA allele. Criteria of %Rank <2 and TPM >1 is used to obtain expressed candidate peptides with strong binding affinities.

We developed a computational pipeline *PREP* to identify A-to-I RE sites from tumor RNA-seq data. The *PREP* workflow consists of two steps (**Figure 1**): A-to-I RE site detection and filtering, candidate RE neoantigen identification. In the first step, RNAEditor (12) was used to detect variants, remove common SNPs, and sequence artifacts to obtain REs. Briefly, raw RNA-seq FASTQ files were processed by trimmomatic version 0.36 (13), reads below an average phred score of 30 were trimmed, then cleaned reads were mapped to reference genome hg38 (ENSEMBL release 83, GRCh38) using STAR (14) (twopassMode), and gene expression was calculated *via* stringtie (15). Duplicated reads were marked and removed by picard-tools version 2.3.0. Indel realignment and base recalibration were conducted by GATK (16) version 3.5 to eliminate false positive variant sites. The GATK Unified Genotyper was utilized to detect all variants with the parameter nucleotide base quality >25 and read mapping quality >20. Three steps were then used to remove common SNP and sequence artifacts, including: (1) remove known genomic variants in dbSNP, 1000 Genome project and HAPMAP project; (2) remove variants in the first and last three base pairs of each read as the edges of sequence reads were error prone; (3) for editing sites in non-Alu regions, intronic variants next to splice junctions were removed as reads might be mapped beyond the corresponding exon boundaries. Variants at the end of homopolymers with at least five repeats were also discarded as sequence errors are likely to occur in these regions. Due to the absence of matched normal RNA-seq data for melanoma cohorts, PREP utilized a ‘panel of normals’ approach to filter out RE sites commonly retained in normal samples, which would not generate immunogenic peptide as a consequence of potential host immune tolerance. REs were identified in six normal skin samples (three individuals, two samples per individual: subject ERS326932 with samples ERR315339 and ERR315376, subject ERS326943 with samples ERR315372 and ERR315460, and subject ERS327007 with samples ERR315401 and ERR315464) from the Human Protein Atlas (**Table S1A**). Paired-end RNA sequencing FASTQ files of all samples were retrieved from the open-access link: https://www.ebiac.uk/arrayexpress/-experiment/E-MTAB-1733/samples/. REs that occurred in at least two samples were classified as normal REs, leading to a final filter set of 135 normal REs (**Table S1B**). These normal REs were removed from REs derived from tumor samples, generating a robust list of putative REs (including Alu sites and non-Alu sites) for each sample. In the second step, all the filtered REs were annotated by Ensembl Variant Effect Predictor (VEP) to obtain non-synonymous REs. We only kept ‘A to G’ substitutions in the plus strand encoding genes or ‘T to C’ substitutions in the minus strand encoding genes. The nucleotide change in non-synonymous RE was translated into the corresponding amino acid change, which was then applied to the proteome reference sequence, leading to a 21-mer peptide containing variant site, and the long peptide was then chopped up into 9–11-mer short peptides (17). HLA allele information was determined from RNA-seq data by OptiType (18). Peptide-MHC binding affinities for both mutant and normal peptides were then inferred by NetMHCpan (version 4.0) (19). Mutant peptide with rank affinity >2% and corresponding gene expression level <1 in transcript per million (TPM) were eliminated.

### Analysis of The Cancer Genome Atlas Pan-Cancer Cohorts

All the A-to-I RE sites of TCGA cohorts were retrieved from Han et al. (20), which identified confident RE sites in 17 cancer types by implementing a series of quality control steps and filters. In brief, RE sites called from RNA-seq data from normal tissues and tumor samples were firstly annotated by ANNOVAR. The sites annotated in dbSNP (version 137), COSMIC, and TCGA somatic mutations were then filtered. On the basis of the RNA-seq reads mapped to the human reference genome (hg19), the editing level at a specific site in a given sample was calculated as the number of edited reads divided by the total number of reads, and only the nucleotides with base quality >=20 were used. Those editing sites with at least three edited reads in at least three samples per tissue type were considered to be detected RE sites. All the A-to-I RE sites were downloaded from Synapse (accession number syn2374375). A-to-I RE sites of 12 cancer types with matched tumor-normal paired information were utilized for A-to-I RE neoantigen analysis. HLA allele information and somatic neoantigen information were downloaded from TCIA database after we obtained the access to the protected TCGA data (phs000178). All the expression data processed by HTseq measured in FPKM were downloaded from TCGA repository. Clinical data of all samples used to perform survival analysis were also retrieved from the TCGA repository. As RE sites of 522 samples across 12 cancer types have matched tumor-normal paired information (**Table S2**), those RE sites having non-zero editing level in every tumor sample and having zero editing level in matched normal sample, termed as “somatic RNA editing site”, were retained, then the second step PREP was applied on these RE sites, leading to the final RE neoantigens for TCGA cohorts. We overlapped the 105 samples in breast cancer from TCGA_RNAediting with 101 breast cancer patients with mass spectrometry data from Mertins et al. (21), leading to 15 breast cancer patients. The neoantigens of these patients were compared with nine highly confidential expressed peptides confirmed by Peng et al. (8) to explore candidate neoantigens. Cancer driver genes were retrieved from two studies related to cancer driver genes of TCGA cohorts (22, 23), which were identified by computational tools such as MutSigCV and OncodriveFM *etc.*, and redundant genes were removed, leading to a list of 977 cancer driver genes (**Table S3**). Cytolytic activity (CYT) was calculated as the geometric mean of *GZMA* and *PRF1* expression in transcripts per million (TPM) (24).

### Ovarian Tumor Analysis With Mass Spectrometry Data of Human Leukocyte Antigen Ligands

Raw RNA-seq data of 14 ovarian tumor samples were retrieved from the National Center for Biotechnology Information Sequence Read Archive project PRJNA398141. *PREP* was performed on these data. *PREP* was run on these epithelial ovarian samples. Mass spectrometric data of these solid tumor samples were obtained from the ProteomeXchange Consortium *via* the PRIDE partner repository by the dataset identifier PXD007635. Raw data (in vendor format) were transformed into mzML format using the command msconvert from ProteoWizard. mzML format of mass spectrometry data of each sample was then searched against a database consisting of 21,042 reviewed protein sequences of the human reference proteome downloaded from Uniprot (release 2018_04) on 11 October 2018 concatenated with putative RE neopeptide using Comet (2018.01 rev. 3). Mass spectrometry search parameters were retrieved from the original study (25); mass tolerance for processing was 0.5 Da for fragment ions and 5 ppm for precursor ions. The only dynamic modification allowed was oxidized methionine, and no cleavage specificity was selected. Peptide confidence was calculated utilizing Percolator (26) with a target value of q <=0.05 (5% FDR) (25).

### Clinical Cohorts Treated With Immune Checkpoint Inhibitors

Analyses were conducted on two independent melanoma cohorts treated with immune checkpoint blockades. The Hugo cohort contained samples from 26 melanoma patients (pt27 was excluded due to abnormal sequence data) treated by the PD-1blockade pembrolizumab (27). Patient outcomes were defined as not responding to therapy (NR) (n = 13) or responding to therapy (R) (n = 13). The Van Allen cohort consisted of pretreatment samples for 40 melanoma patients treated with ipilimumab (3) (anti-CTLA4 therapy). Patient outcomes were defined as not responding to therapy (n = 26) or responding to therapy (n = 14). RNA sequencing of both cohorts was conducted on fresh-frozen tissue utilizing a standard poly(A)-selecting protocol, as described in the original study. Overall survival data utilized in the survival analyses and somatic neoantigen information of both cohorts were also retrieved from the original study.

### Designing of RNA Editing Neoantigen Immunogenicity Score Scheme

Based on our previous study of neoantigen immunogenicity (17), we proposed an efficient score scheme to evaluate the immunogenicity of individual RE neoantigens based on the above features. The individual RE neoantigen immunogenicity *p* was defined as:

(1) $$p=[\tanh(F)\times E][L(R_{m})\times (1-L(R_{n})/2)\times S][H]$$

Where *L* is the logistic function given by:

(2) $$L(x)=\frac{1}{1+e^{5(x-2)}}$$

For paired peptide (mutant peptide and normal peptide) and MHC allele, *R_m_* representing %rank of affinity of the mutant peptide, was obtained by NetMHCpan 4.0 (19); *R_n_* representing %rank of affinity of the normal peptide, was obtained by NetMHCpan 4.0; *F* represents expression level of the mutant gene in Transcript Per Million (TPM); *E* represents editing level of the mutant gene, and editing level was defined as the proportion of edited reads among the total mapped reads at a given position in BAM file; *S* represents sequence dissimilarity between mutant peptide and normal peptide, calculated by 1 minus sequence similarity; *H* represents T cell recognition probability of MHC-peptide determined by peptide hydrophobicity information, which was calculated by a machine learning model proposed in our previous study (17). RE neoantigen immunogenicity score was calculated based on the product of a term representing neo-peptide abundance, a term representing dissimilarity between the mutant peptide and the normal peptide, and a term representing T cell recognition probability. In the first term, a hyperbolic tangent function was applied to the expression level of gene, which is proportional to the expression level at low expression values but asymptotically approaches one at high expression values. RE level was kept with no manipulation. The production of these two metrics represents the abundance of neoantigen. The second term represented potential decrease in immunogenicity of the peptide owing to negative selection against cross-reacting T cells. We applied a sigmoidal logistic function on the rank MHC-peptide binding affinity. This term measures the difference between mutant peptide and normal peptide. The third term was related to T cell recognition probability of MHC-peptide determined by peptide hydrophobicity information. Finally, the overall RE neoantigen immunogenicity score (RENIS) was defined as the sum of all individual RE neoantigen immunogenicity, which was given by:

(3) $$RENIS=\sum p_{i}$$

Based on the overall RE neoantigen immunogenicity score defined above, we further devised the *REScore*, combining neoantigen immunogenicity with infiltrating immune cell population to represent the ability of RE neoantigen to simulate immune response. The *REScore* of each sample was defined as:

(4) REscore=(abundance(CD8)+abundance(CTL))×RENIS

Where RENIS denoted the total RE neoantigen immunogenicity. abundance (*) represents the relative population abundance of immune cell determined by MCPcounter (28) using gene expression data. *CD8* and *CTL* represent CD8^+^ T-cell and cytotoxic lymphocyte respectively.

### Statistical Analysis

We use Wilcoxon rank sum test to assess the difference of ADAR1 expression, RE site burden, and RE neoantigen burden between breast cancer and other cancer types. We use two-sided non-parametric Mann–Whitney *U* test for non-normally-distributed variables (*i.e.*, RE neoantigen burden) to assess the difference in means or median for a continuous variable between two groups (*i.e.*, clinical benefit *vs* no clinical benefit). Spearman’s rank coefficient was utilized to assess the correlation between neoantigen burden and cytolytic score or clinical variates. Spearman’s rank correlation coefficients (SRCCs) were all reported along with corresponding p-value. We utilized the Cox proportional hazard model test and log-rank test to evaluate the correlation between RE neoantigen burden and progression free survival (PFS) in TCGA cohort. We used the Cox proportional hazard model test and log-rank test to assess the correlation between *REscore* and overall survival (OS). We selected the median of each metric as cutoff for high *vs* low group separation in all biomarkers including somatic neoantigen burden (SNB), RE neoantigen burden (RNB), abundance of CD8 T-cell (CD8), abundance of cytotoxic T lymphocytes (CTL) and REscore. All statistical analyses were performed in the R software environment (v3.2.1).

## Results

### Pan-Cancer Study of RNA Editing Neoantigen Profile in The Cancer Genome Analysis Cohort Data

**Figure 2:**
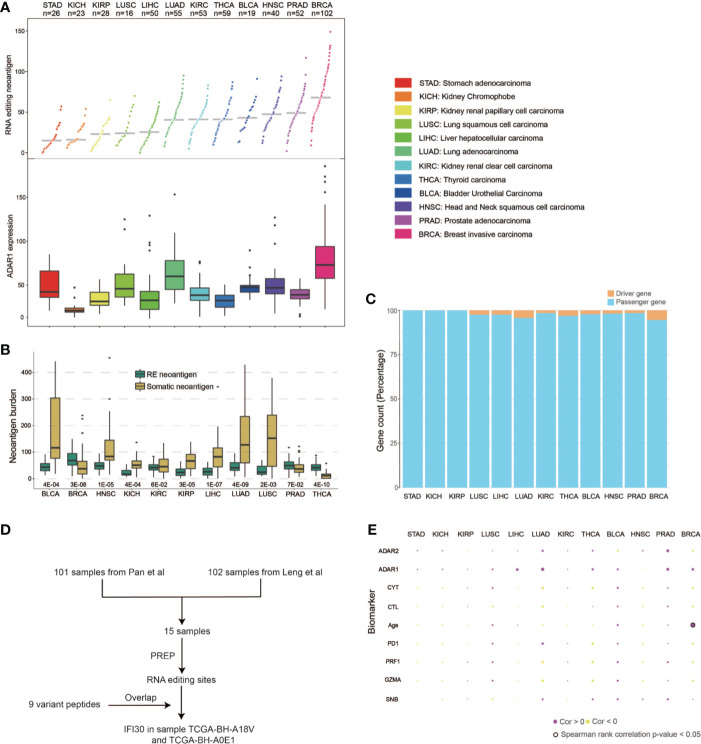
Pan-cancer RE neoantigen profile analysis of TCGA cohorts. **(A)** RE neoantigen distribution and expression of ADAR1 in 12 cancer types. **(B)** RE neoantigen and somatic neoantigen comparison of 12 cancer types. **(C)** Driver gene distribution of neoantigen in each cancer type. **(D)** Flowchart of how IFI30 was confirmed expressed and presented. **(E)** Correlations between RE neoantigen burden and somatic neoantigen burden, expression of CD8A/GZMA/PDCD1/ADAR1/ADAR2 and clinical covariates in 12 cancer types.

We first developed a computational pipeline, *i.e., PREP* (Prioritizing of RNA Editing-based Peptides) to detect the RE neoantigen from tumor RNA-seq data (**Figure 1**). RE events are first identified and filtered, then the variants are applied to the proteomic level to obtain mutant peptides, which are cut into 9–11-mer short peptides. NetMHCpan 4.0 was utilized to predict the binding affinity between the peptide and the given HLA allele. A %Rank <2 and TPM >1 are used to obtain expressed candidate peptides with strong binding affinities (see details in ***Materials and Methods***). We then applied *PREP* to 522 samples (**Table S2**) from 12 cancer types with tumor-normal paired RNA-seq data in TCGA. We identified a median of 68 candidate neoantigen per sample (range from 9 to 149) in breast cancer (**Table S4**), which has a significantly higher RE neoantigen burden compared to those of other cancer types (**Figure 2A**) (Wilcoxon rank sum test p < 0.05 for all, **Table S5A**). This result could be explained as breast cancer has a higher ADAR1 expression, leading to more RE sites than other cancer types (20) (**Figure 2A**) (Wilcoxon rank sum test p < 0.05 for all, **Table S5B**), leading to a higher burden of coding RE sites. We found that breast cancer has a significantly higher RE neoantigen burden than somatic neoantigen burden compared to other cancer types (**Figure 2B**), indicating that RE neoantigen might play a pivotal role in this specific cancer type. However, we found that the ADAR1 expression level was not significantly associated with RE neoantigen burden in all 12 cancer types, nor was the ADAR2 expression level (**Figure 2E**). Cancer driver genes are positively selected along the lineage of cancer development and contribute to the cancer progression; however, only a small portion of RE neoantigens were derived from driver mutation genes (**Figure 2C**) which may be less antigenic, possibly as a consequence of selective pressure by the immune system during tumor development (29, 30). It is noted that Peng et al. (8) have identified 9 A-to-I RE sites with variant peptide support (**Table S6A**) by searching the MS data in 101 breast cancer samples. We found that 15 out of 101 breast cancer samples appeared in previous TCGA breast cohort with paired normal samples. Moreover, analyses on these 15 breast cancer samples resulted in a median of 68 RE neoantigens (range from 9 to 128) per tumor sample (**Table S6B**). Filtered by the nine highly confidential expressed peptides, only protein Gamma-interferon-inducible lysosomal thiol reductase (*IFI30*) was left as our candidate RE neoantigen protein in 10 out of 15 samples, and it was confirmedly expressed in the sample TCGA-BH-A18V and TCGA-BH-A0E1 with MS data support (**Figure 2D**, **Tables S6C, D**). Interestingly, this protein is induced by gamma-interferon in other cell types and expressed constitutively in antigen-presenting cells, playing an pivotal role in MHC class I/II-restricted antigen processing (31). However, its potential to be a candidate neoantigen in breast cancer has not been reported yet.

Previous studies have demonstrated that a high somatic neoantigen burden was associated with significantly longer overall survival in lung adenocarcinoma (LUAD) and other cancer types (32). We first explored whether RE neoantigen burden might be associated with somatic neoantigen burden, expression of the immune markers including *GZMA*/*PRF1*/*PD1*, cytolitic activity (CYT), and clinical covariates in different cancer types. However, there was no significant association between RE neoantigen burden and somatic neoantigen burden in different cancers except in bladder urothelial carcinoma (BLCA), nor was there a correlation with expression of the immune markers including *CD8A*, *GZMA*, *PD1*, or clinical covariates except for the age in breast invasive carcinoma (BRCA) (Benjamini and Hochberg adjusted P > 0.05 for all) (**Figure 2E**, **Table S7A**). Furthermore, we compared the survival predictive power of RE neoantigen burden with other metrics including somatic neoantigen burden, expression of the immune markers and clinical covariates. Both somatic and RE neoantigen burden were not statistically significantly associated with overall survival across different cancer types. There was a significant association between overall survival and immune markers in breast cancer, while no significant associations were observed in other cancer types (**Supplementary Figure 1**, **Table S7B**).

### Validation of RNA Editing Neoantigens in Ovarian Tumor Cohort With Mass Spectrometry Data

**Figure 3:**
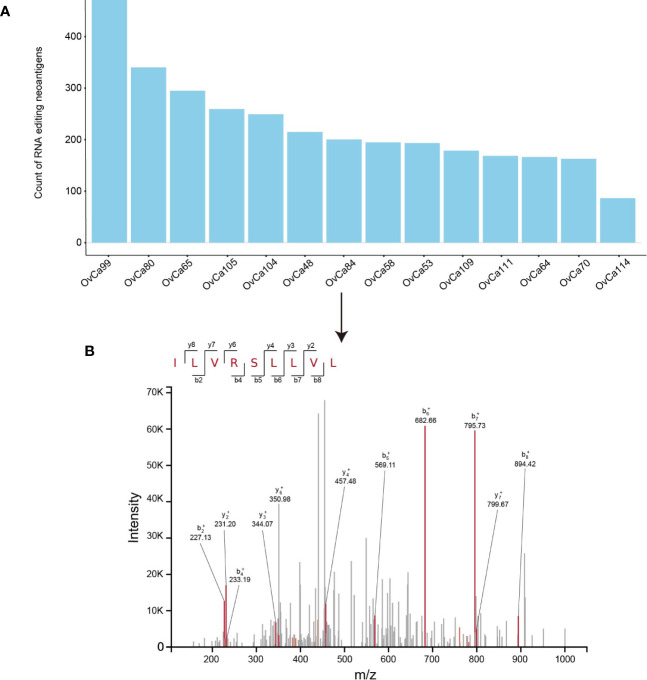
Validation of RE neoantigen in ovarian tumor cohort. **(A)** RE neoantigen identified by PREP in 14 ovarian tumor sample. **(B)** RE neoantigen identified in the OvCa84 tumor sample originating from gene OSBPL9 was predicted by *PREP* and validated by mass spectrometry in the immunopeptidome.

High-resolution mass-spectrometry has facilitated the identification and quantitation of HLA ligands which are naturally processed and presented *in vivo*. This interpretation of the immunopeptidomes involves immunoprecipitation followed by liquid chromatography–mass spectrometry (LC–MS) analysis of the eluted ligands (33). Here *PREP* was applied to 14 ovarian tumor samples with detected neoantigens that were complexed to MHC I by mass spectrometry (25). *PREP* identified a median of 173 candidate neoantigens per tumor (range 76–414) (**Figure 3A**). By searching the mass spectrometry data against database consisting of 21,042 reviewed protein sequences of the human reference proteome downloaded from Uniprot concatenated with putative RE neopeptide, we only confirmed one peptide was processed and presented through the MHC I complex. In OvCa_84 tumor sample, the predicted RE neoantigen ILVRSLLVL from Oxysterol Binding Protein Like 9 (*OSBPL9*) (chr1:51752522) was experimentally discovered in complexed with MHC I *via* mass spectrometry with high confidence (q value <0.05) (**Figure 3B**, **Table S8**). The discovery of RE neopeptide in complexed in MHC I in ovarian cancer sample using mass spectrometry provides further evidence of the processing and presentation of RE neoantigen by the MHC I complex (11).

### Characterization of RNA Editing Neoantigen Profile in Clinical Cohorts With Immunotherapy

**Figure 4:**
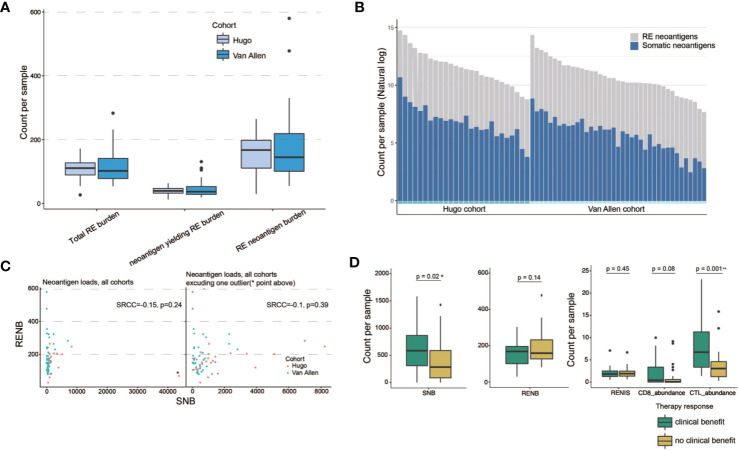
RE neoantigen profiles in clinical cohorts with immunotherapy. **(A)** Distribution of total RE burden, neoantigen-yielding RE burden and RE neoantigen burden in patient cohorts (n = 26 Hugo samples, n = 39 Van Allen samples, two samples were excluded due to lack of somatic neoantigen information). Box plots show the median, first, and third quartiles, whiskers extend to 1.5× the interquartile range. **(B)** Somatic and RE neoantigen burden of individual patient. Within each cohort, patients are sorted by total neoantigen burden. Neoantigen counts (y-axis values) are represented in natural log format. **(C)** Scatterplots show correlation between somatic neoantigen burdens and RE neoantigen burdens, with cohort indicated by color (n = 65 patient samples). One outlier, Hugo_Pt32, indicated on upper plot with asterisks and excluded from the lower plot. **(D)** Association of somatic neoantigen burden (SNB), RE neoantigen burden (RNB), RE neoantigen immunogenicity score (RENIS), and immune cell abundance with clinical benefit from immunotherapy in two melanoma clinical cohorts. Boxplots show the median, first, and third quartiles, whiskers extend to 1.5× the interquartile range and outlier points are plotted individually. Two-side Mann–Whiney U test p-values showed. * denoted significant differential (p < 0.05), ** denoted significant differential (p < 0.01).

**Figure 5:**
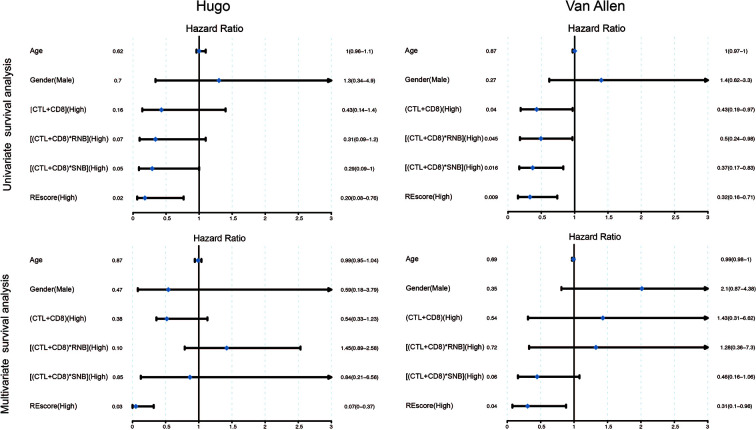
Univariate and multivariate Cox regression survival analysis of combinations of different individual variables in Hugo and Van Allen patient cohorts. The median of each metric was selected as a cutoff for high *vs* low separation in all biomarkers.

We further applied our computational pipeline to tumor sequencing data from two cohorts of melanoma patients treated with immune checkpoint inhibitors (3, 27), as described in *Materials and Methods* (**Tables S9A–B**). Both cohorts have comparable levels of RE sites and predicted RE neoantigens (**Figure 4A**). Minor variation in RE neoantigen burden was observed in consideration of differences in RNA sequencing run, depth, and quality (34). Most patients showed a certain extent of augmented total neoantigen burdens on somatic neoantigen burdens with additional consideration of RE neoantigens. The mean somatic neoantigen burden was 490, and the mean RE neoantigen burden was 144, with the addition of RE neoantigens, producing a ~0.3-fold increase in mean total neoantigen burden (**Figure 4B**). There was no significant correlation between somatic neoantigen burden and RE neoantigen burden (Spearman rank correlation coefficient P = 0.39) (**Figure 4C**). Previous studies have demonstrated that somatic neoantigen burden was significantly correlated with checkpoint inhibitor response in melanoma (3, 27); this conclusion was validated in our study (**Figure 4D**, Wilcoxon rank sum test p = 0.02, samples Pt8 and pat91 were excluded in this analysis as outliers). We next explored whether RE neoantigen burden (RNB) and RE neoantigen immunogenicity (RENIS) might be associated with such response or not. However, there was no significant association between RNB or RENIS and clinical benefit from checkpoint inhibitor therapy (**Figure 4D**, p = 0.14, p = 0.45 respectively). In addition, we examined the relations between immune cell abundance and checkpoint inhibitor therapy response; there exists significant correlation between cytotoxic T lymphocytes (CTL) abundance and therapy response (Wilcoxon rank sum test p = 0.001), while CD8^+^ T-cell abundance does not (Wilcoxon rank sum test p = 0.08). Cytolytic activity score (CYT) has been revealed to be a prognostic biomarker in several cancer types (24, 35); we found that somatic neoantigen burden and RE neoantigen burden were not significantly correlated with the cytolytic activity score, also the clinical covariates including age, gender, disease status and inhibitor status (**Supplementary Figures 2A–F**, Spearman rank correlation coefficient P > 0.05 for all). Finally, we explored the survival predictive power of every single variable including somatic neoantigen burden, RE neoantigen burden, RE neoantigen immunogenicity, cytotoxic T lymphocytes (CTL) abundance, and CD8^+^ T-cell abundance. Both the univariate and multivariate Cox regression analyses revealed that none of these variables showed significant association with improved overall survival (**Supplementary Figure 2G**, log-rank test p > 0.05 for all). As all the variables mentioned above have poor prognoses, we set out to seek a combination for efficient survival prediction. Survival predictive power of four combinations including (CTL + CD8), (CTL + CD8)*SNB, (CTL + CD8)*RNB, (CTL + CD8)*RENIS (defined as REscore, see detailed in *Materials and Methods*) were investigated in two cohorts of melanoma patients. In cohort Hugo, both the univariate and multivariate Cox regression analyses revealed that only high *REscore* showed significant association with improved overall survival. In cohort Van Allen, although univariate Cox regression analysis revealed that all the combinations showed improved overall survival, multivariate Cox regression analysis showed that only REscore is significantly associated with improved overall survival (**Figure 5**). These results indicated that *REscore* indeed captures information related to survival that somatic neoantigen burden and immune infiltrate do not capture and might be an efficient survival predictive biomarker in melanoma patients treated with immune checkpoint inhibitors.

## Discussion

Our analysis of pan-cancer RNA neoantigen profile only covers 12 cancer type, which was subject to the RE information in a previous study. Five cancer types including SKCM were removed as they are lacking of RE sites from normal sample. The predicted neoantigens were unique to cancer cell and would not be subject to central and peripheral tolerance. The RE neoantigen profile analysis reported that only breast cancer exhibited tremendous difference between somatic neoantigen burden and RE neoantigen burden. It could be explained and verified by previous researches that breast cancer generally does not carry a high mutation burden, leading to low somatic neoantigen burden (36), whereas A-to-I RE is a major source of mRNA variability in breast cancer. This phenomenon suggested that RE neoantigen might be a better choice as therapeutic target of immunotherapy including cancer vaccine and adoptive T-cell therapy for breast cancer or other cancer types with low somatic neoantigen burden.

We identified one peptide out of 2,795 that expressed RE sites in 14 ovarian tumor samples, which seems to account for a low proportion. This discrepancy between the number of RE sites observed and HLA-bound peptides detected is in the range of other proteogenomics studies which also report less than 1% of the transcriptomic sites or genomic sites to be presented in peptide number. For example, Minying Zhang et al. (11) identified three out of 1,369 RE sites (0.21%), Bassani-Sternberg et al. (37) identified 11 out of 3,487 somatic mutations (0.32%). This discrepancy might be affected by biological factor and LC–MS sensitivity like TAP and binding affinity to HLA, cytosolic peptidases or proteasome processing.

One limitation of the PREP pipeline is that although we implemented a series of procedures to eliminate the false positive editing sites as much as possible, the false positives still remain. The identified RE neoantigen burden using PREP is about three times more than that from the TCGA cohort (median of 170 in ovarian and melanoma cohorts *versus* median of 68 in TCGA cohort). One reason might be that the tools and parameters for identification and filter were different between TCGA pipeline and PREP, which might result in differences of final editing sites. Another important reason may be that our pipeline did not filter out germline RE event as we did not have matched normal RNA-seq data in the latter three cohorts, incorporation of matched normal tissue will improve elimination of germline RE sites with the increased prediction accuracy.

In summary, we systematically characterize the A-to-I RE neoantigens in 12 cancer types. We reported that RE neoantigen might play a pivotal role in breast cancer and *IFI30* might be a potential as therapeutic target. The *REscore* we proposed showed a strong correlation with expression of the immune markers, and a higher *REscoFdatare* was associated with a significantly longer overall survival in clinical cohorts of melanoma undergoing immune checkpoint inhibitors therapy. Taking together, the exploration of patient-specific RE neoantigen improves our understanding of cancer neoantigen and provides more types of candidates for personalized cancer vaccines designed in the context of cancer immunotherapy.

## Data Availability Statement

The original contributions presented in the study are included in the article/**Supplementary Materials**, further inquiries can be directed to the corresponding authors.

**Code availability.** *PREP* is available at https://github.com/bm2-lab/PREP.

## Author Contributions

QL and ZY conceived the study. CZ, ZW and ZY analyzed the tumor sample data. LZ and ZY provided useful discussions on RNA editing related topics. CZ, ZY and QL wrote the manuscript with assistance from other authors. All authors contributed to the article and approved the submitted version.

## Funding

This work was supported by the National Key Research and Development Program of China (Grant No. 2017YFC0908500, No. 2016YFC1303205), National Natural Science Foundation of China (Grant No. 31970638, 61572361), Shanghai Natural Science Foundation Program (Grant No. 17ZR1449400), Shanghai Artificial Intelligence Technology Standard Project (Grant No. 19DZ2200900) and Fundamental Research Funds for the Central Universities.

## Conflict of Interest

The authors declare that the research was conducted in the absence of any commercial or financial relationships that could be construed as a potential conflict of interest.
