# Supplementary material for: Systematically Characterizing A-to-I RNA Editing Neoantigens in Cancer
Source: Front Oncol. 2020 Dec 10;10:593989. doi: 10.3389/fonc.2020.593989 (PMC7758481; doi:10.3389/fonc.2020.593989)
Supplement: Supplementary file 10 [file DataSheet_10.docx]

**Supplementary informations**

**Systematically Characterizing A-to-I RNA Editing Neoantigens in Cancer**

Chi Zhou^1, #^, Zhiting Wei^1, #^, Liye Zhang^3^, Zhaoyi Yang^2,*^, Qi Liu^1,*^

^1^ Translational Medical Center for Stem Cell Therapy and Institute for Regenerative

Medicine, Shanghai East Hospital, Bioinformatics Department, School of Life Sciences

and Technology, Tongji University, Shanghai 200092, China.

^2^Department of Pharmacy, The First Affiliated Hospital of University of Science and Technology of China, Hefei, China.

^3^School of Life Science and Technology, ShanghaiTech University, Shanghai, China.

# Contribute equally

Correspondence: Qi Liu; [qiliu@tongji.edu.cn](mailto:qiliu@tongji.edu.cn)

Zhaoyi Yang; young2382@163.com

**Figure S1** Univariate Cox regression survival analysis of 9 biomarkers including RE neoantigen burden (RNB), somatic neoantigen burden (SNB), expression of immune markers, cytolytic activity (CYT), cytotoxic T lymphocyte abundance (CTL) and clinical covariates (age and gender) in 12 cancer types.

**.**


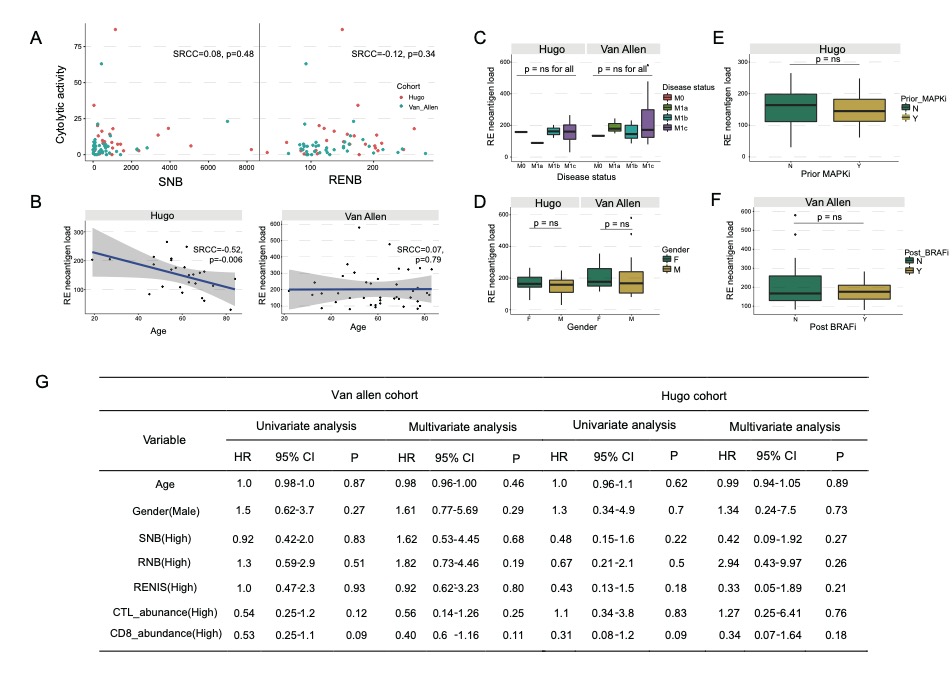


**Figure S2** **Association between** RE **neoantigen burden (RNB) and patient clinical characteristics.** (A) Correlation between somatic neoantigen burden (SNB), RE neoantigen burden (RNB), cytolytic activity. Spearman rank corraltion and corresponding p value showed. (B) Association between RE neoantigen burden (RNB) and age. (C) Association between RE neoantigen burden (RNB) and disease status. (D) Association between RE neoantigen burden (RNB) and gender. (E) Association between RE neoantigen burden (RNB) and MAPKi therapy status in Hugo cohort. (F) Association between RE neoantigen burden (RNB) and MAPKi therapy status in Van Allen cohort. Two-side Mann-Whiney U test p-values > 0.05. (G) Univariate and multivariate Cox regression survival analysis somatic neoantigen burden (SNB), RE neoantigen burden (RNB), RE neoantigen immunogenicity score (RENIS) and immune cell abundance (cytotoxic T lymphocytes and CD8+ T cells) in Hugo and Van Allen patient cohorts. SNB: Somatic neoantigen burden; RNB: RE neoantigen burden; RENIS: RE neoantigen immunogenicity score; CTL: Cytotoxic T lymphocyte
